# Is There a “Black Friday” for Geriatric Hip Fracture Surgery?

**DOI:** 10.1111/os.13741

**Published:** 2023-04-13

**Authors:** Chuwei Tian, Huanyi Zhu, Liu Shi, Xiangxu Chen, Tian Xie, Yunfeng Rui

**Affiliations:** ^1^ Department of Orthopaedics, Zhongda Hospital, School of Medicine Southeast University NO.87 Ding Jia Qiao Nanjing 210009 PR China; ^2^ Multidisciplinary Team (MDT) for Geriatric Hip Fracture Management, Zhongda Hospital, School of Medicine Southeast University Nanjing PR China; ^3^ Orthopaedic Trauma Institute (OTI) Southeast University Nanjing 210009 PR China; ^4^ Trauma Center, Zhongda Hospital Southeast University Nanjing 210009 PR China; ^5^ School of Medicine Southeast University NO. 87 Ding Jia Qiao Nanjing 210009 PR China

**Keywords:** Adverse Outcomes, Delayed Surgery, Friday Admission, Hip Fracture, Risk Factors

## Abstract

**Objectives:**

Reports show an increase in the short‐term mortality rates of hip fracture patients admitted on weekends. However, there are few studies on whether there is a similar effect in Friday admissions of geriatric hip fracture patients. The aim of this study was to evaluate the effects of Friday admission on mortality and clinical outcomes in elderly patients with hip fractures.

**Methods:**

A retrospective cohort study was performed at a single orthopaedic trauma centre and included all patients who underwent hip fracture surgery between January 2018 and December 2021. Patient characteristics, including age, sex, BMI, fracture type, time of admission, ASA grade, comorbidities, and laboratory examinations, were collected. Data pertaining to surgery and hospitalization were extracted from the electronic medical record system and tabulated. The corresponding follow‐up was performed. The Shapiro–Wilk test was applied to evaluate the distributions of all continuous variables for normality. The overall data were analyzed by Student's t test or the Mann–Whitney U test for continuous variables and the chi‐square test for categorical variables, as appropriate. Univariate and multivariate analyses were used to further test for the independent influencing factors of prolonged time to surgery.

**Results:**

A total of 596 patients were included, and 83 patients (13.9%) were admitted on Friday. There was no evidence supporting that Friday admission had an effect on mortality and outcomes, including length of stay, total hospital costs and postoperative complications. However, the patients admitted on Friday had delayed surgery. Then, patients were regrouped into two groups according to whether surgery was delayed, and 317 patients (53.2%) underwent delayed surgery. The multivariate analysis showed that younger age (*p* = 0.014), Friday admission (*p* < 0.001), ASA classification III‐IV (*p* = 0.019), femoral neck fracture (*p* = 0.002), time from injury to admission more than 24 h (*p* = 0.025), and diabetes (*p* = 0.023) were risk factors for delayed surgery.

**Conclusions:**

Mortality and adverse outcome rates for elderly hip fracture patients admitted on Friday were similar to those admitted at other time periods. However, Friday admission was identified as one of the risk factors for delayed surgery.

## Graphical abstract

Mortality and adverse outcome rates for geriatric hip fracture patients admitted on Friday were similar to those admitted at other time periods. However, Friday admission was identified as one of the risk factors for delayed surgery.

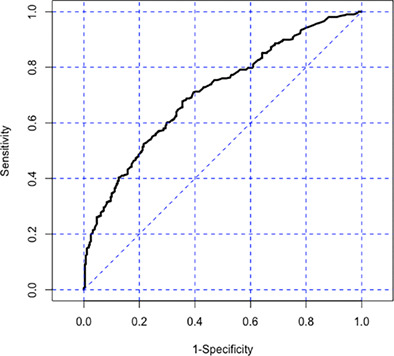

## Introduction

Hip fracture is one of the most serious fall‐related injuries among elderly patients in the public health system. Compared to other fractures, geriatric hip fracture has a worse prognosis, more complications, a higher mortality rate, and a higher cost.^1^, ^2^ In China, hip fracture has become an increasingly important public health problem because of the aging population.^3^ Furthermore, geriatric hip fracture is associated with decreased mobility and decreased quality of living as well as increased family dependence, increased demand for social health services and mental or financial burdens.^2^, ^4^

At present, many studies have analyzed factors that influence the mortality of hip fracture. Most of these studies focus on patient characteristics, but the influence of medical conditions has been ignored.^5^, ^6^ On account of hip fracture being a serious emergency, an event occurring during the weekend will pose a challenge to the healthcare system. The “weekend effect” has been the subject of recent debate and is linked to a variety of medical conditions, including head trauma, stroke, myocardial infarction, and ruptured aortic aneurysm.^7^, ^8^, ^9^, ^10^, ^11^, ^12^, ^13^, ^14^ Whether similar weekend effects occur in patients with hip fractures remains uncertain. Recently, studies on the weekend effect on patients with hip fractures show conflicting results.^15^, ^16^, ^17^, ^18^, ^19^ These results may be due to different levels of medical development and different guidelines in different regions.

Friday, however, is a neglected time node. Patients admitted on Fridays were also affected by inadequate medical resources and delayed surgery when compared with patients admitted on weekends. A multicentre study found that the Dutch hospitals showed a “Friday effect” for elective surgery, with an adjusted odds ratio (OR) of 1.33 for 30 days death compared to Monday admission.^20^ At the same time, we found that most studies have underscored the importance of timely surgery. Ackermann et al. considered delaying surgery no more than 48 h if necessary.^21^ In clinical practice, for patients admitted on Fridays, surgery is often postponed until the Monday of the following week if they do not have surgery over the weekend to treat the hip fracture. This leads to a higher risk of delayed surgery for geriatric patients with hip fractures admitted on Fridays. However, there are few studies on the effect of Friday admission on the preoperative waiting time of geriatric hip fracture patients.

This study focused on analyzing the effect of Friday admission on short‐term mortality, clinical outcomes, and postoperative complications in elderly patients with hip fracture. The aims of the retrospective study were as follows: (i) whether patients with geriatric hip fractures admitted on Fridays have an increase in the short‐term mortality rates and (ii) to analyze the reasons for delayed surgery due to Friday admission.

## Patients and Methods

### Study Design, Setting, and Population

A retrospective study was performed at a single orthopaedic trauma centre, Zhongda Hospital, Nanjing, China, from January 2018 to November 2021. This study was approved by the institutional board review of Zhongda Hospital Affiliated to Southeast University (2022ZDSYLL183‐P01).

The inclusion criteria were: (1) age older than 60 years at the time of injury; (2) a confirmed diagnosis of hip fracture; (3) postoperative follow‐up performed at least 30 days.

The exclusion criteria were: (1) admitted with hip osteoarthritis or fractures around the prosthesis; (2) received conservative treatment due to severe comorbidities; (3) with missing data.

**FIGURE 1:**
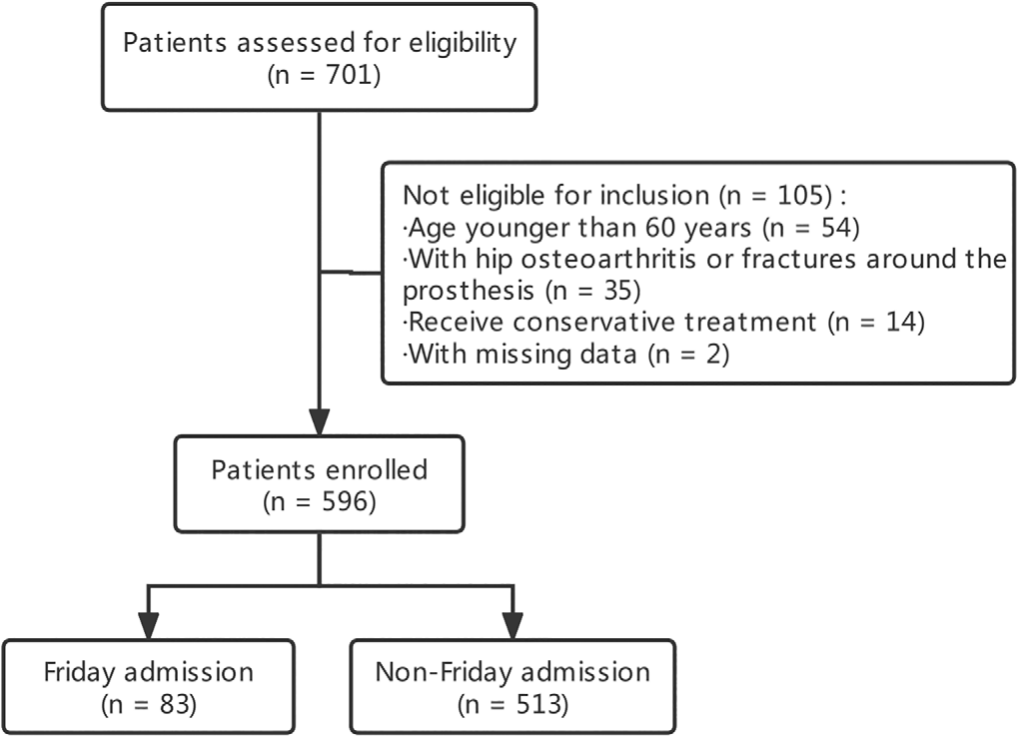
Flow diagram of the patients included in this research.

The patients were retrospectively assigned to two groups according to their admission day: Friday admission (n = 83, 13.9%) and non‐Friday admission (n = 513, 86.1%). The flow diagram of the included patients in this research is shown in Figure 1.

### Perioperative Treatment and Surgical Procedure

Our center provides 24 h support on the weekend, including national holidays with at least two orthopaedic surgeons in the ward daily. We have a multidisciplinary team including geriatricians, anaesthesiologists, and intensive care unit (ICU) physicians, to review perioperative care for patients with comorbidities.^22^ Furthermore, our multidisciplinary team has established an ICU fast‐track for elderly patients with hip fractures, which allowed patients in poor general condition to be immediately transferred to the ICU for intensive care.^23^

During the perioperative period, all patients received a comprehensive evaluation and standardized diagnosis and treatment. The patients who underwent surgical treatments were operated on by the same team. The patients with femoral neck fractures underwent surgical treatments such as total hip arthroplasty (THA), hip hemiarthroplasty (HHA), or multiple screws fixation. The patients with an intertrochanteric fracture underwent surgical treatments such as intramedullary nail fixation (Gramma 3). After the operation, the patients were encouraged to perform early partial‐to‐full weight‐bearing with the necessary assistance of others. Discharged patients were followed up by a call or visit to the orthopaedic clinic.

### Data Collection

The data were retrospectively collected from the electronic patient records at the institution by two orthopaedic surgeons. Demographic data included sex, age, body mass index (BMI), and general health status according to the American Society of Anaesthesiologists (ASA) classification^24^, ^25^ and comorbidities (consisting of hypertension, diabetes, acute coronary syndromes, history of fracture, and thrombogenesis). The injury‐related data included fracture type (femoral neck fracture or intertrochanteric fracture), time from injury to admission, and laboratory examinations at admission. The surgery‐related data included type of surgery, type of anesthesia, use of ICU fast‐track, time to surgery, and duration of surgery (THA, HHA, multiple screws and intramedullary nail fixation). The in‐hospital data included total hospital costs (THC), length of stay (LOS), short‐term postoperative complications, and laboratory examinations after surgery. The follow‐up data included patients' survival status and date of death. The follow‐up started after surgery, and the endpoint events were defined as all‐cause mortality or end of study, whichever was earlier. All patients were followed‐up for at least 90 days.

### Definitions

Patient admission days were categorized into Friday and Saturday to Thursday. Patient's BMI was classified as malnutrition with BMI < 18.5 kg/m^2^, normal with 18.5 ≤ BMI < 24 kg/m^2^, overweight with 24 ≤ BMI < 28 kg/m^2^, and obesity with BMI ≥28 kg/m^2^.^26^ Time to surgery was defined as the time from admission to the hospital to surgery. Delayed surgery was defined as an operation performed more than 48 h after admission. ASA classification was grouped as I‐II and III‐IV. Short‐term postoperative complications were defined as those that occurred during hospitalization and follow‐up. In addition, the time of surgery was used as a scale for the analysis of mortality.

### Outcome Measures

The primary outcomes of this study included short‐term postoperative mortality, while the secondary outcomes included preoperative waiting time, surgery duration, postoperative time in the hospital, LOS, THC, short‐term postoperative complications, and laboratory examination after surgery.

All patients were followed up from discharge until the date of death or the end of the study. Each patient was followed up for at least 90 days. Time, cause of death, 30 days all‐cause mortality, and 90 days all‐cause mortality were recorded. Short‐term postoperative complications are classified as pulmonary complications (consisting of pulmonary infection and respiratory failure), urinary complications (consisting of urinary infection, acute kidney injury, and organ injury), neurological complications (consisting of transient ischaemic attack and delirium), and cardiovascular or cerebrovascular complications (consisting of acute myocardial infarction, acute cerebral infarction, heart failure, ischaemic heart disease, low cardiac output, and cardiac dysfunction). The follow‐up results were based on the outpatient clinical system and telephone contact with patients and their family members.

### Statistical Analysis

We evaluated the distributions of all continuous variables for normality by using the Shapiro–Wilk test. Data satisfying normalcy are presented as the mean and standard deviation (SD). Nonnormally distributed variables are presented as the median (IQR). Categorical variables are shown as counts (percentages). The overall data were analyzed by Student's t test or the Mann–Whitney U test for continuous variables and the chi‐square test for categorical variables, as appropriate. Univariate and multivariate analyses were used to further test for the independent influencing factors of prolonged time to surgery. All statistical analyses were performed using SPSS statistical software (version 26.0, SPSS Inc., Chicago, USA). A two‐sided *p* value <0.05 was considered significant.

## Results

### Population and Patient Characteristics

The baseline characteristics of the patients are shown in Table 1. A total of 596 patients were enrolled in the final analysis; the patients were divided into Friday (n = 83) and non‐Friday (n = 513) groups based on the time of admission. There were no statistically significant differences between the two groups in terms of sex, age, BMI, ASA classification, fracture type, time from injury to admission, comorbidities, laboratory examination at admission, type of surgery, type of anesthesia, or use of ICU fast‐track.

**TABLE 1:** Baseline patient characteristics. Values are the count (%) unless otherwise specified

Variables	Friday	Non‐Friday	Statistic	*p* value
	n = 83	n = 513		
Sex			χ^2^ = 1.164	0.281
Male	31 (37.3)	161 (31.4)		
Female	52 (62.7)	352 (58.6)		
Age, Mean **±** SD, years	80.83 ± 8.46	80.67 ± 8.07	t = 0.163	0.870
BMI, Mean **±** SD, kg/m^2^	22.35 ± 3.32	22.70 ± 3.71	t = −0.813	0.416
Malnutrition	14 (16.9)	63 (12.3)	χ^2^ = 3.354	0.340
Normal	45 (54.2)	270 (52.6)		
Overweight	21 (25.3)	137 (26.7)		
Obesity	3 (3.6)	42 (8.4)	t = −0.813	0.416
ASA classification			χ^2^ = 2.141	0.143
I‐II	71 (85.5)	403 (78.6)		
III‐IV	12 (14.5)	110 (21.4)		
Fracture type			χ^2^ = 1.500	0.221
Femoral neck fracture	38 (45.8)	272 (53.0)		
Intertrochanteric fracture	45 (54.2)	241 (47.0)		
Time from injury to admission			χ^2^ = 1.449	0.229
≤ 24 h	71 (85.5)	410 (79.9)		
>24 h	12 (14.5)	103 (20.1)		
Number of comorbidities, Mean **±** SD	3.73 ± 1.88	3.86 ± 2.21	t = −0.486	0.627
Hypertension	47 (56.6)	295(57.5)	χ^2^ = 0.023	0.881
Diabetes	17 (20.5)	133 (25.9)	χ^2^ = 1.124	0.289
ACS	27 (32.5)	189 (36.8)	χ^2^ = 0.575	0.448
History of fracture	33 (39.8)	195 (38.0)	χ^2^ = 0.092	0.761
Thrombogenesis	14 (16.9)	99 (19.3)	χ^2^ = 0.275	0.600
Laboratory examination at admission				
RBC, Mean **±** SD	3.75 ± 0.66	3.79 ± 0.68	t = −0.492	0.623
WBC, Mean **±** SD	9.04 ± 2.94	9.50 ± 3.47	t = −1.128	0.260
Hb, Mean **±** SD	115.95 ± 19.82	117.64 ± 19.80	t = −0.720	0.472
ALB, Mean **±** SD	38.15 ± 4.15	38.13 ± 5.01	t = 0.025	0.980
PT, Mean **±** SD	11.90 ± 1.14	12.40 ± 6.60	t = 0.697	0.486
APTT, median [IQR]	29.6 [26.8–32.1]	30.0 [27.8–32.4]	Z = −1.021	0.307
INR, median [IQR]	1.09 [1.04–1.17]	1.11 [1.06–1.19]	Z = −1.332	0.183
Type of surgery			χ^2^ = 4.353	0.226
THA	17 (20.5)	105 (20.5)		
HHA	17 (20.5)	154 (30.0)		
intramedullary nail fixation	45 (54.2)	241 (47.0)		
multiple screws fixation	4 (4.8)	13 (2.5)		
Type of anesthesia			χ^2^ = 0.232	0.630
General	76 (91.6)	461 (89.9)		
Regional	7 (8.4)	52 (10.1)		
ICU fast‐track	21 (25.3)	134 (26.1)	χ^2^ = 0.025	0.875

### Friday Admission Did Not Increase Short‐Term Mortality but Led to Delayed Surgery

For the primary outcomes, a total of 596 patients were followed up for at least 90 days after surgery. For 30 days mortality, one patient (1.2%) was lost to follow‐up in the Friday group, and 10 (1.9%) patients in the non‐Friday group were lost to follow‐up. For 90 days mortality, two patients (2.4%) were lost to follow‐up in the Friday group, and 13 (2.5%) in the non‐Friday group were lost to follow‐up. Table 2 shows that there was no significant difference in 30 and 90 days mortality between the two groups (*p* > 0.05).

**TABLE 2:** Comparison of outcomes. Values are the count (%) unless otherwise specified

Variables	Friday	Non‐Friday	Statistic	*p* value
	n = 83	n = 513		
**Primary outcome**				
30‐day mortality^a^	0/82 (0.0)	14/503 (2.8)	χ^2^ = 2.338	0.237
90‐day mortality^b^	1/81 (1.2)	30/300 (6.0)	χ^2^ = 3.134	0.077
**Secondary outcome**				
Time to surgery, median [IQR]	75.1 [64.9–92.8]	46.8 [37.6–82.8]	Z = −5.435	*p* < 0.001
≤48 h	11 (13.3)	268 (52.2)	χ^2^ = 43.617	*p* < 0.001
>48 h	72 (86.7)	245 (47.8)		
Duration of surgery, median [IQR]	90.0 [79.8–120.0]	94.8 [80.0–115.0]	Z = −0.423	0.672
Time after surgery, median [IQR]	7.15 [5.87–8.92]	7.07 [6.03–9.63]	Z = −0.198	0.843
LOS, median [IQR]	10.91 [8.02–12.97]	9.79 [7.98–12.68]	Z = −1.564	0.118
THC, median [IQR]	55547.14 [46536.68–70983.17]	58833.54 [49386.01–69255.98]	Z = −0.669	0.503
Short‐time postoperative complications	25 (28.9)	158 (30.8)	χ^2^ = 0.015	0.901
Pulmonary infections	10 (12.0)	60 (11.7)	χ^2^ = 0.009	0.926
Urinary infections	0 (0.0)	6 (1.2)	χ^2^ = 0.981	0.405
Neurological complications	10 (12.0)	45 (8.8)	χ^2^ = 0.915	0.339
Cardiovascular or cerebrovascular	3 (3.6)	42 (8.2)	χ^2^ = 2.140	0.143
Laboratory examination, Mean ± SD				
Hb after surgery	98.45 ± 13.70	99.27 ± 14.74	t = −0.471	0.638
Hb variation	−17.65 ± 17.62	−18.54 ± 17.61	t = 0.425	0.671
ALB after surgery	33.08 ± 3.53	33.05 ± 3.55	t = −0.054	0.957
ALB variation	−5.05 ± 5.93	−5.11 ± 5.08	t = −0.087	0.930

^a^ 11 data missing, 1 of Friday and 10 of Non‐Friday

^b^ 15 data missing, 2 of Friday and 13 of Non‐Friday.

For the secondary outcomes, we found that the patients admitted on Friday had longer times before surgery than those admitted between Saturday and Thursday (median 76 h vs 47 h, *p* < 0.001). The difference in delayed surgery between the two groups was still statistically significant (87% vs 48%, *p* < 0.001). There were no significant differences in residual factors, including surgery duration, postoperative time in hospital, length of stay (LOS), total hospital costs (THC), short‐term postoperative complications, and laboratory examination after surgery, between the two groups (*p* > 0.05).

### Risk Factors for Delayed Surgery

**FIGURE 2:**
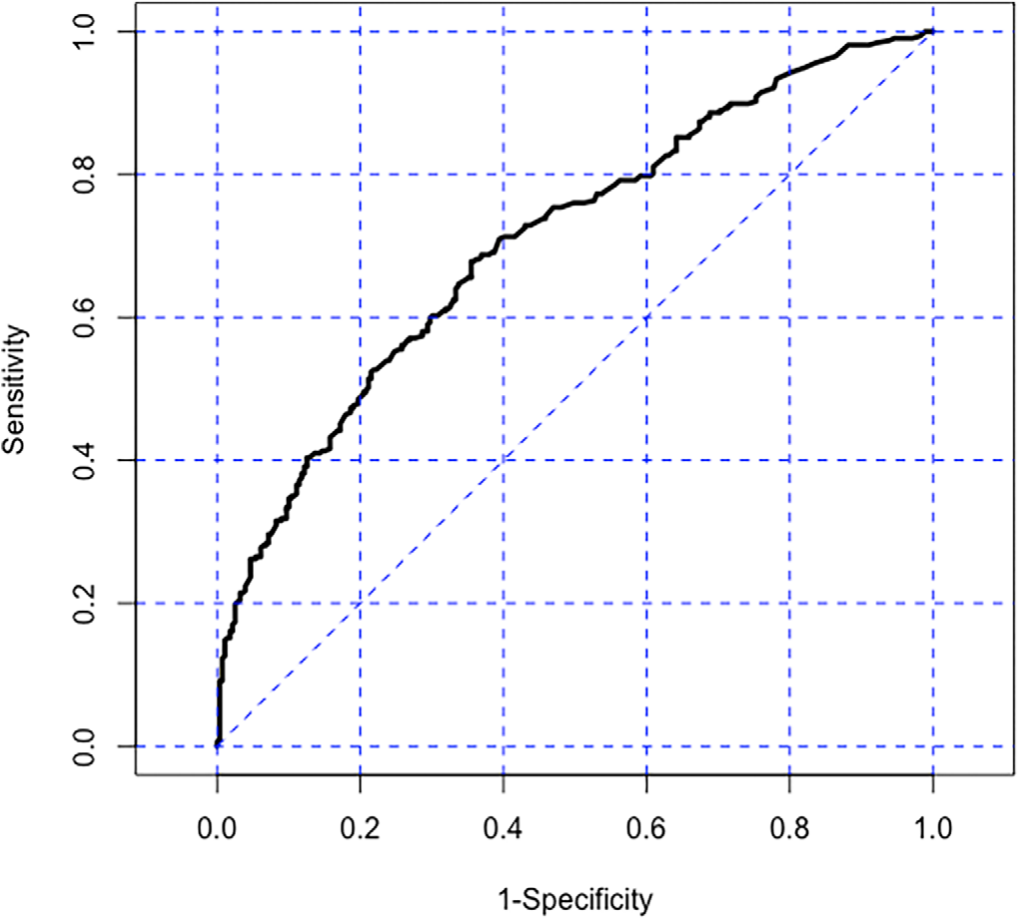
ROC for the multivariate log regression analysis, AUC = 0.708 (95% CI 0.667–0.749).

A total of 596 patients were divided into two groups based on whether surgery was delayed (279 vs. 317). The baseline characteristics and univariate analysis of these patients are shown in Table 3. The univariate analysis showed that seven variables, including age, Friday admission, ASA III‐IV, femoral neck fracture, time from injury to admission more than 24 h, number of comorbidities and diabetes, met the criteria for inclusion in the logistic regression model (*p* < 0.05). The results from the multivariate logistic analysis are presented in Table 4. The multivariate logistic analysis showed that younger patients (vs. older patients, OR 0.972, 95% CI 0.951–0.994, *p* = 0.014), Friday admission (vs. non‐Friday admission, OR 9.069, 95% CI 4.613–17.829, *p* < 0.001), ASA III‐IV (vs. ASA I‐II, OR 1.686, 95% CI 1.089–2.610, *p* = 0.019), femoral neck fracture (vs. intertrochanteric fracture, OR 1.8, 95% CI 1.2–2.5, *p* = 0.002), time from injury to admission more than 24 h (vs. less than 24 h, OR 1.665, 95% CI 1.067–2.599, *p* = 0.025), and diabetes (vs. without diabetes, OR 1.598, 95% CI 1.068–2.391, *p* = 0.023) were risk factors for delayed surgery. The number of comorbidities was excluded from the model. The ROC analysis for the multivariate log regression showed that the AUC was 0.708 (95% CI 0.667–0.749) (Figure 2).

**TABLE 3:** Univariate analysis of factors associated with delayed surgery. Values are the count (%) unless otherwise specified

Variables	Time to surgery ≤48 h	Time to surgery >48 h	Statistic	*p* value
	n = 279	n = 317		
Sex			χ^2^ = 1.067	0.302
Male	84 (30.1)	108 (34.1)		
Female	195 (69.9)	209 (65.9)		
Age, Mean ± SD, years	81.77 ± 7.84	79.75 ± 8.26	t = −3.051	0.002
BMI, Mean ± SD, kg/m^2^	22.35 ± 3.77	22.91 ± 3.55	t = 1.893	0.059
Malnutrition	37 (13.3)	40 (12.6)	χ^2^ = 1.702	0.636
Normal	154 (55.2)	161 (50.8)		
Overweight	68 (24.4)	90 (28.4)		
Obesity	20 (7.2)	26 (8.2)	t = 1.893	0.059
Friday admission	11 (3.9)	72 (22.7)	χ^2^ = 43.617	*p* < 0.001
ASA classification			χ^2^ = 4.232	0.040
I‐II	232 (83.2)	242 (76.3)		
III‐IV	47 (16.8)	75 (23.7)		
Fracture type			χ^2^ = 10.927	0.001
Femoral neck fracture	125 (44.8)	185 (58.4)		
Intertrochanteric fracture	154 (55.2)	132 (41.6)		
Time from injury to admission			χ^2^ = 6.060	0.014
≤ 24 h	237 (84.9)	244 (77.0)		
> 24 h	42 (15.1)	73 (23.0)		
Number of comorbidities, Mean ± SD	3.62 ± 1.94	4.03 ± 2.34	t = 2.319	0.021
Hypertension	164 (58.8)	178 (56.2)	χ^2^ = 0.420	0.517
Diabetes	58 (20.8)	92 (29.0)	χ^2^ = 5.341	0.021
CHD	49 (17.6)	48 (15.1)	χ^2^ = 0.638	0.424
ACI	100 (35.8)	116 (36.6)	χ^2^ = 0.036	0.849
History of fracture	108 (38.7)	120 (37.9)	χ^2^ = 0.046	0.830
Thrombogenesis	53 (19.0)	60 (18.9)	χ^2^ = 0.000	0.983
Laboratory examination at admission				
RBC, Mean ± SD	3.78 ± 0.66	3.79 ± 0.68	t = 0.315	0.753
WBC, Mean ± SD	9.51 ± 3.42	9.37 ± 3.40	t = −0.519	0.604
Hb, Mean ± SD	116.90 ± 19.52	117.85 ± 20.01	t = 0.588	0.557
ALB, Mean ± SD	38.30 ± 5.50	37.99 ± 4.30	t = −0.768	0.433
PT, Mean ± SD	12.05 ± 1.63	12.60 ± 8.31	t = −1.101	0.271
APTT, median [IQR]	30.2 [27.7–32.4]	29.7 [27.7–32.3]	Z = −0.640	0.522
INR, median [IQR]	1.11 [1.06–1.19]	1.11 [1.05–1.19]	Z = −0.063	0.950

**TABLE 4:** Multivariate logistic regression analysis

Outcome parameter	β	Wald	*p* value	Exp (β)	95% CI
Age	−0.28	6.048	0.014	0.972	(0.951, 0.994)
Friday admission	2.205	40.876	*p* < 0.001	9.069	(4.613, 17.829)
ASA classification III‐IV	0.522	5.490	0.019	1.686	(1.089, 2.610)
Femoral neck fracture	0.576	9.979	0.002	1.778	(1.244, 2.541)
Time from injury to admission >24 h	0.510	5.042	0.025	1.665	(1.067, 2.599)
Diabetes	0.469	5.198	0.023	1.598	(1.068, 2.391)

## Discussion

In our study, we found that Friday admission did not affect the length of stay, postoperative short‐term complications, 30 days mortality, or 90 days mortality among patients with hip fractures. Furthermore, the multivariable analysis showed that younger age, Friday admission, higher ASA classification, femoral neck fracture, time from injury to admission more than 24 h, and diabetes were independent risk factors for delayed surgery.

### Friday Admission Did Not Result in a Worse Outcome

Despite advances in healthcare quality and medical interventions, older patients with hip fractures have worse outcomes compared with younger patients after surgery. It has been reported that 10% of hip fracture patients die within 30 days of surgery.^27^, ^28^, ^29^ Studies by Bell et al. and Freemantle et al. reported no difference in mortality between weekday and weekend admissions.^8^, ^30^ In contrast, some studies have shown that weekend admission affects the outcomes of hip fracture patients. The existence of this contradiction is related to whether the regional medical system is developed or not and the guidelines. According to Foss et al. and Thomas et al., weekend admission was associated with a significant increase in 30 days mortality among hip fracture patients.^31^, ^32^ In addition, Ruiz et al. examined the records of 28 hospitals in England, Australia, the United States, and the Netherlands and found higher odds of 30 days death for weekend emergency admissions.^20^ Among them, the Dutch hospitals showed a significant “Friday effect” for elective surgery, with an adjusted OR of 1.33 for 30 days death compared to Monday admission. Clague et al. found that while Friday admission was a predictor of 90 days mortality in patients with hip fractures, it did not have an effect on in‐hospital mortality.^17^ In addition, some studies have confirmed that delaying surgery leads to a higher risk of pneumonia.^33^ However, in our study, although patients admitted on Friday had a higher risk of delayed surgery, the difference in the probability of postoperative pneumonia between the two groups was not statistically different. The results of these studies were inconsistent with our study. This might be due to different treatments for patients. Our hospital has a multidisciplinary team, which was fully staffed on weekends and holidays to provide adequate support to patients, and the staff is able to review the perioperative care for patients with comorbidities.^22^ This might be the cause of our findings.

### Friday Admission Led to Delayed Surgeries

Notably, several studies have found that Friday and weekend admissions lead to delayed surgeries, which in turn are associated with higher rates of death and complications.^16^, ^34^, ^35^, ^36^ In regard to the risk factors associated with delayed surgery, some studies reported that different admission days, higher ASA grade, longer time from injury to admission, and diabetes were the factors influencing preoperative waiting time, which was consistent with our study.^16^, ^36^, ^37^, ^38^ In addition, we found that relatively young elderly patients were more likely to experience delays before undergoing surgery. The reason may be related to Moran et al.'s reports that delaying surgery up to 4 days did not increase postoperative mortality in relatively young patients.^34^ We also found that a femoral neck fracture was a risk factor. It has been reported that the average age of patients with femoral neck fractures is approximately 2 years younger than that of patients with intertrochanteric fractures, suggesting that patients with femoral neck fractures are in better physical condition.^39^, ^40^ In contrast, patients with intertrochanteric fractures had severe pain and more hidden blood loss, which was the reason for early surgery. In 2011, Fantini et al. showed that INR >1.5 and arrhythmia were risk factors for delayed surgery, which was not consistent with the results of our study.^16^ This might be caused by the progress of surgical technology, anesthesia level, anesthesia drugs, and differences in ward management. Our multidisciplinary team allowed for a more comprehensive assessment of the patient's overall condition, and the ICU fast‐track could help patients smoothly pass through the perioperative period.^22^, ^23^

The weekend effect is due to several factors, such as limited availability of experienced staff and surgeon fatigue.^41^, ^42^ These variables vary widely between healthcare systems and can influence the outcomes of patients with life‐threatening and complex emergencies. Patients are less affected by reduced staffing because of the clear diagnosis and limited pre‐ and postoperative care required for hip fracture.^31^

There are many reasons for the delay of surgery due to Friday admission, and the main reason is that surgery is rarely performed on weekends. The guidelines recommend that patients with hip fractures undergo surgery within 48 h.^15^, ^19^ Patients admitted on Friday must wait longer than 48 h if surgery cannot be performed on the weekend. In contrast, patients admitted on weekends might have shorter surgery waiting times. Of course, the patient's comorbidities and fracture type were also important risk factors for delayed surgery. The significance of this study was to further optimize the allocation of medical resources on weekends and shorten the waiting time of patients before surgery.

Moreover, the health care system in China differs from that in developed Western countries because most of the hospitals in China are open year‐round and perform emergency surgeries on weekends. Our findings are consistent with previous studies showing that Friday admission had no effect on short‐term postoperative mortality but that Friday admission resulted in delayed surgery. In addition, since the establishment of a multidisciplinary team at our hospital in 2018, the rate of 48 h surgery for older patients with hip fractures has increased from 27.6% (2016–2018) to 46.8% (2018–2021), even though our results still show that patients admitted on Friday are more likely to have delayed surgery. In addition, our multidisciplinary team can fully assess the patient's status before surgery, and an ICU fast track allows for slow resuscitation under supervision and early functional exercise after surgery. This may be the reason for the similar prognosis between the two groups in our study.

### Strengths and Limitations

There are some strengths to our study. First, this is the first study to specifically investigate the effects of Friday admission on short‐term mortality, postoperative complications, and delayed surgery in patients with hip fractures in China. Second, we analyze risk factors for delayed surgery in patients with hip fractures. These results suggest that we should pay attention to patients who are relatively young, have femoral neck fractures, are complicated with diabetes, and are admitted on Fridays in clinical work. With these results, clinicians can quickly identify high‐risk populations for delayed surgery in geriatric patients with hip fracture following multidisciplinary treatment. The preoperative assessment and diagnosis process would be further optimized under the MDT model to reduce the preoperative waiting time of patients.

Our study has the following limitations. First, it is a retrospective study with the possibility of missing data. Second, our study is a single‐centre study, all of the patients were from level 1 trauma centres, and the patients had more severe comorbidities and advanced disease than those treated in the community. Third, we have a multidisciplinary team for hip fractures and have access to surgery all year round, including weekends, which is not available in some hospitals and medical facilities. Thus, selection bias can be expected. The results of this study need to be further verified and generalized by multi‐centre and large sample studies.

### Conclusion

In summary, the study demonstrates that geriatirc patients with hip fractures admitted on Friday have similar mortality and adverse outcome rates compared with elderly patients with hip fractures admitted at other time periods. However, Friday admission was identified as one of the risk factors for delayed surgery. The significance of this study is that it can help orthopaedic surgeons further optimize the diagnosis and treatment process of geriatric hip fractures by using a multidisciplinary team and by appropriately scheduling surgery, and this can allow better treatment of patients during the perioperative period.

## Author Contributions

CWT designed this study and wrote the manuscript. HYZ performed the experiments and analyzed the data. CWT and HYZ collected the clinical data. LS, XXC, and TX provided technical support. YFR provided the idea, revised and proofread the paper. All authors read and approved the final manuscript.

## Ethical Statement

This current study was approved by the institutional board review of Zhongda Hospital Affiliated to Southeast University (2022ZDSYLL183‐P01). Written informed consent for participation was not required for this study in accordance with the national legislation and the institutional requirements.

## Conflicts of Interest

On behalf of all authors, the corresponding author states that there is no conflict of interest.
